# Native Hawaiian and Pacific Islanders’ Identity and Housing Status: The Impact on Historical Trauma and Perceived Stress

**DOI:** 10.3390/ijerph21091249

**Published:** 2024-09-21

**Authors:** Tessa Palafu, Danielle L. Carreira Ching, Veronica M. Acosta, Scott K. Okamoto, Kelsie H. Okamura

**Affiliations:** 1 The Baker Center for Children and Families, Harvard Medical School, Boston, MA 02120, USAkokamura@jbcc.harvard.edu (K.H.O.); 2 Department of Psychology, Hawai‘i Pacific University, Honolulu, HI 96813, USA; 3 Population Sciences in the Pacific Program, University of Hawai‘i Cancer Center, Honolulu, HI 96813, USA; 4 Department of Psychology, University of Hawai‘i at Mānoa, Honolulu, HI 96822, USA

**Keywords:** historical trauma, Native Hawaiian or Pacific Islander health, homeless youth, subjective stress, health status disparities, resilience

## Abstract

Native Hawaiian and Pacific Islanders (NHPIs) are overrepresented in Hawai‘i’s houseless population. Indigenous populations, such as NHPIs, may encounter experiences of historical trauma that impact their well-being. This original research project examines how NHPI identity and houselessness compound to affect the perceived stress and historical trauma of transition-aged youth. Fifty-one participants aged 18 to 24 (*M* = 21.37, *SD* = 1.93) completed a survey that included the historical traumatic events scale, historical loss scale, perceived stress scale, and a demographic questionnaire. Over half (*n* = 26, 51.0%) of the participants identified as NHPI. A two-way ANOVA indicated a non-significant effect of NHPI identity and housing status on perceived stress. However, housed participants scored significantly higher than participants experiencing houselessness on the historical traumatic events scale (*p* = 0.006). Our findings elucidate the role of knowledge in the experience of historical trauma. Further results, limitations, and future directions are offered.

## 1. Introduction

Native Hawaiian and Pacific Islanders (NHPIs) are overrepresented in Hawai‘i's houseless population, accounting for only 10% of the state’s overall population, but making up 35% of the state’s houselesss (This study uses the term houselessness to classify individuals experiencing homelessness. Based on community engagement with this population, individuals have negative associations with the word “homelessness.” Thus, to respect participants' views, this study will use the term houselessness.)population [1]. The experience of houselessness presents physical and mental health risks to people, including food and water insecurity and increased barriers to healthcare [2]. A study following 288 HIV+ men experiencing houselessness found that unmet hygiene and structural needs had the strongest negative effect on their self-reported mental health [3]. People overrepresented in the houseless population, such as NHPIs, also face unique health-related risks (e.g., higher rates of hypertension and heart disease) that worsen their well-being compared to non-NHPI people [4]. Health disparities in NHPIs are a pervasive issue, with suicide being the leading cause of death in NHPI aged 15–24 in 2019 [5] and Native Hawaiian women experiencing the highest breast cancer mortality rates in Hawai‘i [6]. These higher disease and death rates are consistent with what is observed in other Indigenous populations, namely American Indian and Alaskan Natives [7].

Some scholars have characterized the persistent health disparities seen across Indigenous communities as a link to the historically traumatic events experienced by multiple generations [8,9]. The experience of these events and the constellation of the responses to them (e.g., increased depression, suicide, and substance use rates) have been coined historical trauma [8], which is defined as the “collective complex trauma inflicted on a group of people who share a specific group identity or affiliation” [9], p. 320. This encompasses both the experience and response to traumatic events. Similar to other Indigenous populations, foreign colonial powers have impacted NHPI communities, leaving lasting political, economic, and health-related legacies. Therefore, the health disparities observed across NHPI communities may be explained as a response to the historically traumatic events experienced by these communities (e.g., the illegal overthrow of the Hawaiian monarchy, nuclear testing in the Marshall Islands). In Hawai‘i, Riley and colleagues described Native Hawaiian youth’s (aged 15–24) historical trauma as a common feeling of “Kaumaha” or heavy and oppressive sadness due to the cumulative losses of land, sovereignty, and family from early death [10]. Additionally, another study of Native Hawaiian college students found that historical trauma correlated with increased perceived discrimination and substance use [11]. This limited research suggests an emerging relationship between historical trauma and overall well-being that begins in childhood.

Another important consideration for NHPI houselessness is the experience of chronic stress. Studies have shown that chronic stress exposure from early childhood through to young adulthood may disrupt typical developmental trajectories and result in adverse health outcomes [12] and is a risk factor for the development of psychopathology (e.g., post-traumatic stress disorder, anxiety, depression) [13]. There is limited research on the specific link between NHPI houselessness and stress; however, qualitative studies offer insights into the daily experiences of houseless NHPI youth and families. Interviews with houseless NHPIs reveal frustrations with government policies that impact housing availability, experiences of domestic violence, and the absence of a sense of safety [14,15]. Such experiences could cause significant distress. Additionally, studies have found that individuals from low SES backgrounds may exhibit significant cognitive differences (e.g., changes in their brain surface area) from individuals from high SES backgrounds due to a persistent state of stress, which could have long-term impacts on multiple facets of their life, including educational achievement [16]. Once again, research on the specific impact of poverty on the cognitive development of NHPIs is limited; however, early studies suggest that poverty impacts the overall health of NHPI populations [17,18]. The intersection of chronic stress and houselessness at critical developmental periods can have long-term impacts on lifetime trajectories, including altering the function of the body’s stress systems, which can have negative implications for physical and mental health [12]. For example, research has shown that exposure to chronic stressors during early life may induce abnormal reactivity in a part of the brain that partially facilitates bodily and behavioral responses to the environment, which has been linked to memory deficits and mental and physical health concerns [12]. If houseless NHPI populations have higher levels of chronic stress due to historical trauma and other stressors, this could result in higher rates of adverse health conditions than in other populations.

Taken together, it is critical to examine the extent to which houselessness and NHPI identity impact the experience of historical trauma and perceived stress in transition-aged youth (ages 18–24). We hypothesize that (a) NHPI transition-aged youth will have higher levels of historical trauma compared to their non-NHPI counterparts, (b) transition-aged youth experiencing houselessness will have higher levels of historical trauma compared to their housed counterparts, and (c) NHPI transition-aged youth experiencing houselessness will have higher levels of perceived stress than all other groups (housed NHPIs, housed non-NHPIs, and non-NHPIs experiencing houselessness).

## 2. Materials and Methods

### 2.1. Participants

Participants were recruited through community-based organizations that serve houseless populations and local universities. There were 72 participants who completed our informed consent form. In total, 21 participants were excluded due to exclusionary criteria (*n* = 15) and data integrity concerns (*n* = 6; see the data analytics plan). Specifically, 15 participants were excluded because they were (a) outside of the age range of 18–24 (*n* = 7) or (b) did not provide their age (*n* = 8). The remaining 51 participants were included in the analysis. Participants had an average age of 21.37 (*SD* = 1.93) years (see Table 1). Most participants identified as Asian (*n* = 30; 58.8%), female (*n* = 32; 62.7%), and housed (*n* = 32; 62.7%). A total of 26 (51.0%) participants identified as NHPI, 20 of whom are Native Hawaiian. Most participants report high school as their highest education level (*n* = 14; 27.5%), have a yearly income of USD 0–15,000 (*n* = 21; 41.2%), have never experienced food insecurity (*n* = 20; 39.2%), and have access to mental health services (*n* = 32; 62.7%).

### 2.2. Measures

***Historical traumatic events scale [19].*** This eight-item scale adapted for use in Native Hawaiian populations examines the generational experience of historically traumatic events [11]. Participants indicated whether the provided events (e.g., being forced not to speak their native language or practice cultural expression) were experienced by themselves, their parents, grandparents, great-grandparents, great-great-grandparents, none, or not sure. The total score was calculated by summing all positive responses across the eight items and its reliability, as measured by internal consistency, was within an acceptable range (α = 0.74).

***Historical loss scale [20].*** This eight-item scale asked participants to indicate how often (i.e., never, yearly or at special occasions, monthly, weekly, daily, several times a day, or don’t know) they think about the provided experiences (e.g., a loss of traditional spiritual ways). The total score was calculated by taking the sum across the eight items and its reliability, as measured by internal consistency, was in the excellent range (α = 0.88).

***Perceived stress scale [21].*** This 10-item survey asked participants how often they experienced stress-related symptoms (e.g., felt nervous or stressed) in the last month. Responses ranged from never (0) to very often (4). The total score was calculated by summing across the 10 items, with scores less than 13 indicating low stress, scores of 14–26 indicating moderate stress, and scores greater than or equal to 27 indicating high perceived stress. Its reliability, as measured by internal consistency, was in the acceptable range (α = 0.73).

***Demographic form.*** This 10-item demographic form asked the participants’ age, ethnicity, primary ethnic identity, housing status, gender identity, highest level of education achieved, annual income, experience of food insecurity, and access to mental health services.

### 2.3. Procedure

Participants were recruited with the help of 13 community agencies that service low-income individuals and the houseless population and university departments within the state of Hawai‘i. The research team initiated contact with organization partners via email, detailing the project’s goals and process. Requests for zoom meetings were included to answer any questions. From there, partner organizations were able to either (a) forward the request to participate in the study to their students or clients and/or (b) request that a member of the research team distribute the surveys in-person. If agencies chose in-person, a member of the research team would work with their organization, distribute surveys, and answer participant questions in real time.

Prior to participating in the study, participants read and acknowledged an informed consent form and then completed the brief survey (10 min or less) either online (via REDCap) or in-person. A USD 10 gift card was given to anyone who completed or attempted to complete the survey. For in-person distribution, participants received their incentive on the spot. Participants that completed the survey online were either mailed a physical incentive or received a digital incentive, based on their preference. The contact number for the Hawai‘i CARES line was provided in case the survey’s content was triggering or the participant’s perceived stress score was at a moderate–high level (perceived stress scale score > 14). The Hawaiʻi Pacific University institutional review board approved this study.

### 2.4. Data Analytics Plan

The various housing and ethnicity variables were combined to form four simple groups: (a) NHPI (Fijian, Guamanian or Chamorro, Marshallese, Micronesian, Native Hawaiian, Palauan, Samoan, Tahitian, or Tongan), (b) non-NHPI (all other groups), (c) housed (living with friends, living with family, and in permanent housing), and (d) houseless (outdoor public area, emergency shelter, and transitional shelter). Participants were able to select more than one ethnicity and if they selected any ethnicity that fell within the NHPI category, they were included in the NHPI group. To further prepare the data, the research team examined missing responses and implemented listwise deletion to preserve data integrity, which led to six additional participants being excluded from the analyses. These listwise deletions were due to an incomplete historical traumatic events scale (*n* = 1), no information on the historical loss scale (*n* = 3), and an incomplete perceived stress scale (*n* = 2). The team then examined the distribution of the scales’ (historical traumatic events, historical loss, and perceived stress scale) means and standard deviations. These results were consistent with other studies that have used these scales.

All data analyses were carried out with SPSS v28. Descriptive analyses were run for the demographic information (see Table 1). A 2 × 2 ANOVA was used to test the hypothesis that NHPIs experiencing houselessness would score higher on the perceived stress scale than all other groups (housed NHPIs, housed non-NHPIs, and non-NHPIs experiencing houselessness). Pairwise comparisons using estimated marginal means and a Least Significant Difference adjustment for multiple comparisons were used to determine the differences in perceived stress within subgroups. Two-sample t-tests were conducted to compare NHPIs and non-NHPIs and houseless versus housed people on the historical loss and historical traumatic events scales. Alpha was set at 0.05 to detect significant differences between these groups.

## 3. Results

### 3.1. The Interaction between NHPI Identity and Houselessness on the Perceived Stress of Transition-Aged Youth

**Figure 1:**
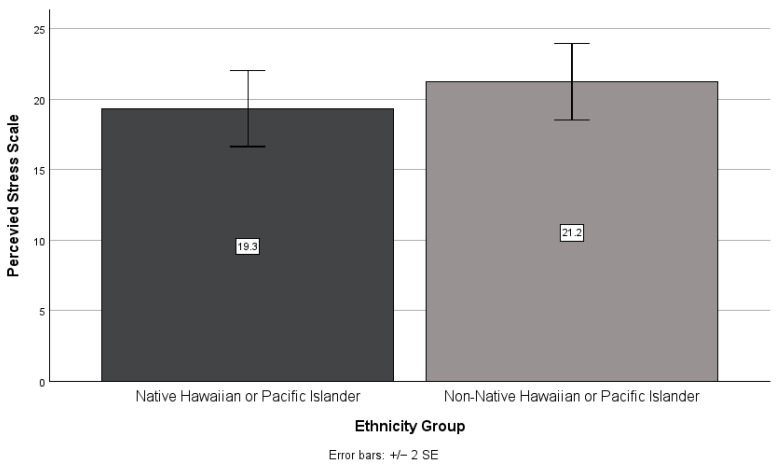
NHPIs versus non-NHPIs’ mean perceived stress scale scores.

**Figure 2:**
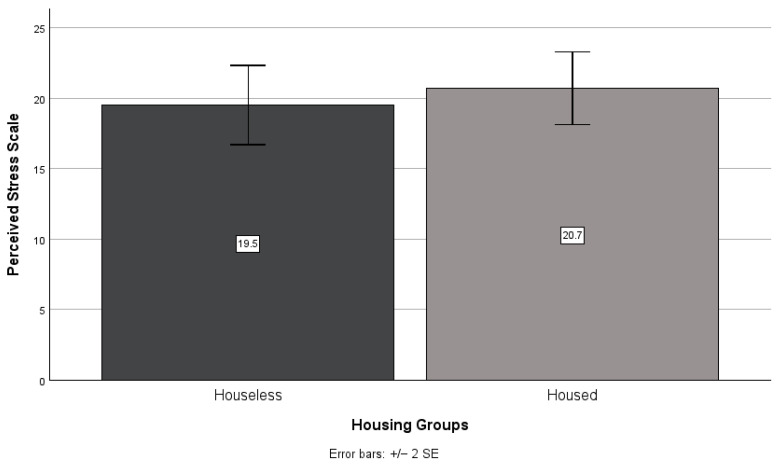
Houseless versus housed participants’ mean perceived stress scale scores.

There was no significant interaction between the effects of NHPI identity and houselessness on perceived stress scores (*F*(1,1) = 0.08, *p* = 0.776). Pairwise comparisons showed that NHPI identity (F(1,47) = 1.00, *p* = 0.322, see Figure 1) and houselessness (F(1,47) = 0.31, *p* = 0.579, see Figure 2) did not have a significant effect on perceived stress. Non-NHPIs scored higher than NHPIs (*M* = 21.2, *SD* = 6.8 and *M* = 19.3, *SD* = 6.9, respectively), and housed higher than houseless (*M* = 20.7, *SD* = 7.2 and *M* = 19.5, *SD* = 6.1, respectively), on the perceived stress scale.

### 3.2. Houselessness, Historical Trauma, and Perceived Stress

**Figure 3:**
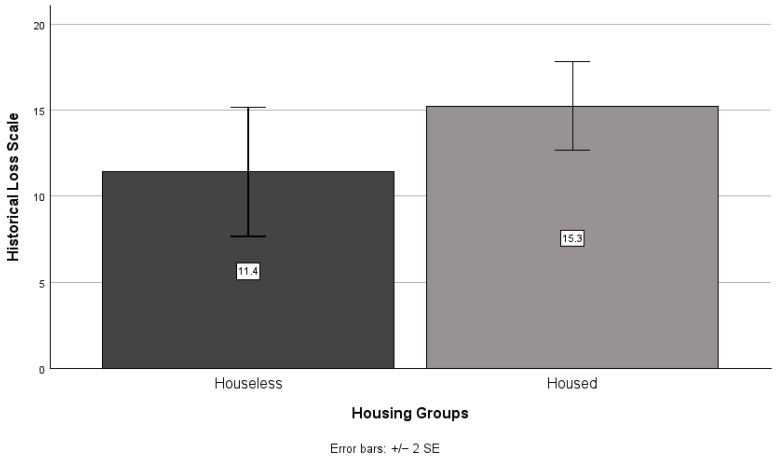
Houseless versus housed participants’ mean historical loss scale scores.

**Figure 4:**
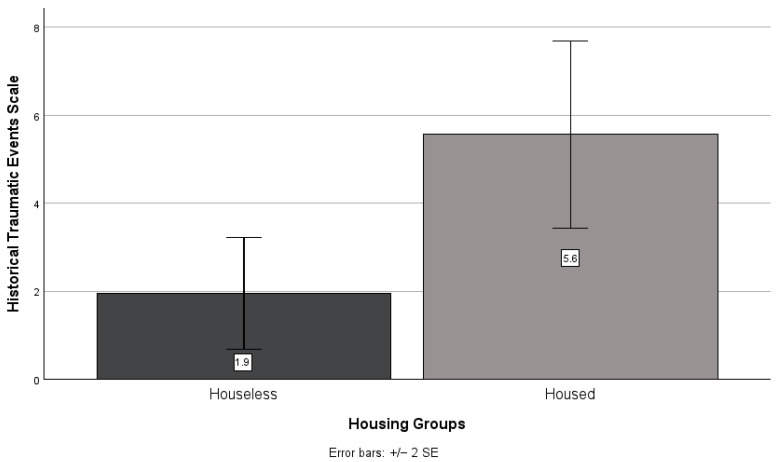
Houseless versus housed participants’ mean historical traumatic events scale scores.

There was no significant difference between houseless and housed participants’ scores on the historical loss (*t*(49) = −1.74, *p* = 0.089; see Figure 3) scale. However, housed participants scored higher than houseless participants on the historical loss (*M* = 15.3, *SD* = 7.2 and *M* = 11.4, *SD* = 8.2, respectively) scale. There was a significant difference between housed and houseless participant scores on the historical traumatic events scale (*t*(46.818) = −2.91, *p* = 0.006; see Figure 4), with housed participants (*M* = 5.6, *SD* = 6.0) exhibiting higher scores than houseless participants (*M* = 1.9, *SD* = 2.8).

### 3.3. NHPI Identity, Historical Trauma, and Perceived Stress

**Figure 5:**
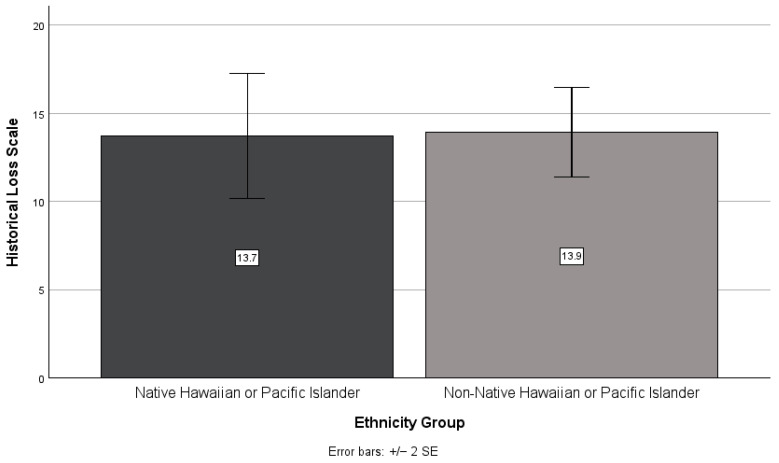
NHPIs versus non-NHPIs’ mean historical loss scale scores.

**Figure 6:**
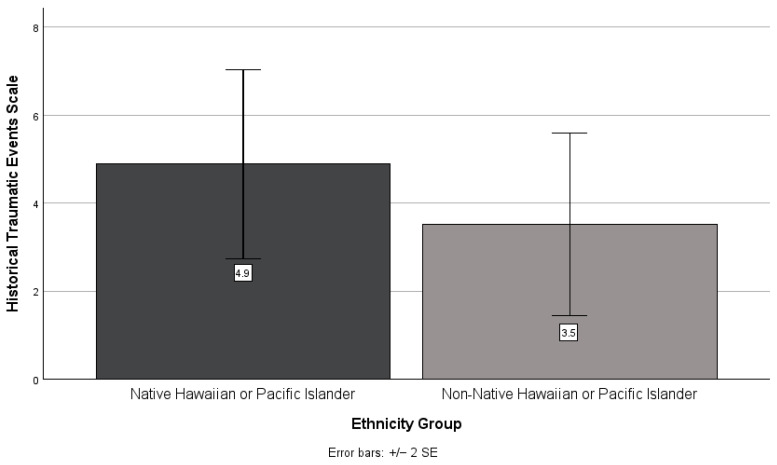
NHPIs versus non-NHPIs’ mean historical traumatic events scale scores.

There was no significant difference between NHPI and non-NHPI participant scores on the historical loss (*t*(49) = −0.09, *p* = 0.932; see Figure 5) and historical traumatic events (*t*(49) = 0.91, *p* = 0.366; see Figure 6) scales. However, non-NHPI participants scored higher than NHPI participants on the historical loss scale (*M* = 13.9, *SD* = 6.4 and *M* = 13.7, *SD* = 9.0, respectively), whereas NHPI participants scored higher than non-NHPI participants on the historical traumatic events scale (*M* = 4.9, *SD* = 5.5 and *M* = 3.5, *SD* = 5.2, respectively).

## 4. Discussion

This study examined the impact of NHPI identity and houselessness on the perceived stress and historical trauma experienced by transition-aged youth. Contrary to our hypotheses, there were no significant interactions between the effects of NHPI identity and houselessness on transition-aged youths’ perceived stress score. The literature on the role of perceived discrimination [11,22,23] and population-specific strengths [15,24] give context to these unexpected results. The results did reveal significant historical traumatic event score differences between housed and houseless participants, where housed participants scored higher on the historical traumatic events scale, which was also contrary to the hypotheses, but may be explained by the disruption of knowledge transfer in houseless populations [25]. There were also no differences in historical trauma, historical loss, and perceived stress between NHPI and non-NHPI participants. This is contrary to the study hypotheses, but the impact of historical events on non-NHPI groups [26,27] and the role of culture in NHPI well-being [28,29,30,31] should be considered. These differential findings within transition-aged youth prompt a reconsideration of how houselessness and NHPI identity interact with well-being.

There was a significant difference between housed and houseless participants’ scores on the historical traumatic event scale. Housed participants reported more personal and generational historical traumatic events experienced, which does not support our hypothesis. The previous literature has not examined the use of the historical traumatic events scale within a houseless population; however, research has shown that Indigenous groups tend to score higher on this scale than other populations [19]. One explanation for the findings related to the historical traumatic events scale is the differential access to knowledge. Knowledge is a privilege, and the historical traumatic events scale focuses on the experience of the past six generations; knowledge that may not be easily accessible or well known. Oftentimes, such knowledge is passed down through family members, particularly in Indigenous populations. However, those experiencing houselessness may have experienced family separation prior to their current living situation. For instance, Shaikh and Rawal interviewed individuals experiencing houselessness in northern Canada and found that many of the participants described experiencing family separation from a young age due to a variety of reasons, including early family death, relocation through the child welfare system, and being forcefully sent to boarding schools [25]. Family separation at a young age can disrupt that critical knowledge transfer, especially among Indigenous populations. Post hoc analyses showed a positive association between degree status and scores on the historical traumatic events scale. Participants with higher-education degrees were more likely to report experiences of historical traumatic events by their parents (*p* = 0.022), grandparents (*p* = 0.001), great-grandparents (*p* < 0.001), and great-great-grandparents (*p* = 0.003). This reveals that knowledge, as measured by degree-holding status in this example, could play a role in the experience of historical trauma. Future studies should look to explicitly examine the role of knowledge in the experience of historical trauma. Additionally, a closer examination on the impact of family separation on knowledge transfer amongst individuals experiencing houselessness, and particularly in Indigenous communities, is warranted.

Related to perceived stress, it is important to reconsider the role of NHPI identity in perceived stress levels. This study proposed that those who endorsed NHPI identity would score higher than those who did not due to the experience of historical and modern-day stressors. However, other non-NHPI demographic groups may also face similar stressors, both in terms of content and frequency, which could be reflected in these findings [26,27]. This categorization could have skewed the results by underestimating the impact of similar experiences on different historically oppressed groups. Therefore, ethnic categorization alone may not be the variable from which differences in perceived stress emerge. Future studies should examine the role other variables (e.g., the frequency of stressors, distance from historically traumatic events, news coverage, etc.) play in the differences seen in the ways historically marginalized groups perceive stress.

Culture, as a protective factor, may explain the unexpected results related to a lack of significant differences between NHPIs and non-NHPIs on the historical traumatic events and historical loss scales. Indigenous communities have led discourse on the importance of cultural connection in promoting the well-being of Indigenous groups. Discussions with American Indian and Alaskan Native groups highlight how a connection to culture (e.g., values, identity, practices, community connection) serves as a source of resilience when confronted with oppressive systems, racist behaviors, and the impacts of historical trauma [28,29]. For example, an Inupiaq elder described how their spirituality helped them cope with being away from their home [28]. A recent study found that Native Hawaiians who report more positive feelings regarding their Native Hawaiian identity also report higher life satisfaction [30]. Similarly, a study looking at Pacific Islander well-being in Hawai‘i found that biculturalism (i.e., the combining and practicing of customs from two nations, peoples, or ethnic groups) was associated with higher self-esteem, which has impacts on healthy eating and well-being [31]. These findings suggest that cultural identity could disrupt the expected relationship between NHPI identity and historical trauma by serving as a source of resilience. Future studies should examine the role that culture plays in the experience of historical trauma within NHPI populations.

The role of perceived discrimination (i.e., perceived unfair treatment) could also provide some context for these perceived stress results by acting as a mediator. One study examining the relationship between historical trauma and substance use in Native Hawaiian college-aged students found that perceived ethnic discrimination (i.e., perceived unfair treatment on a day-to-day basis) mediated the association between historical trauma and substance use [11]. Research with descendants of Indian Boarding School attendees found that those who considered their Indigenous background a central component of their identity were more likely to detect and perceive discriminatory situations [22]. Furthermore, a recent study found that NHPI ethnicity had an indirect effect on mental health symptomology and substance use behaviors via perceived discrimination [23]. Therefore, there may not be a direct effect of NHPI identity and the experience of houselessness on perceived stress levels; rather, there could be indirect pathways mediated by variables not presently measured, such as perceived discrimination.

The presence of protective factors could also explain the non-significant result of the interaction between NHPI identity and houselessness on perceived stress. Although this study explored the potential impact of risk factors on stress, community strengths were not examined. A recent study that interviewed previously houseless NH and Micronesian families found that receiving support from other houseless families, taking initiative, deriving meaning from their situation, and spirituality/morality (e.g., connecting to a higher power, chasing after their goals) all served as protective factors [15]. Another project with Indigenous youth experiencing houselessness found that hope, resilience, and optimism were positively associated with life satisfaction [24]. Although NHPI youth experiencing houselessness encounter daily stressors, the presence of these protective factors may lessen the impact of these events on their perceived stress levels. Future studies should conduct interviews with houseless NHPI youth to understand the factors that support their well-being.

Various limitations should be considered in the interpretation of these results. The small sample size limited the type of analyses conducted and, therefore, the results, and caution should be exercised when generalizing the findings. Based on the results of comparisons between ethnicity, housing, and combined groups, the average effect size was 0.16, indicating a small, but significant, effect. Previous research has indicated that distrust towards researchers within NHPI, and other Indigenous, communities due to historical injustices serve as a barrier to recruitment [32,33]. Community-based participatory research (i.e., “a collaborative research approach that is designed to ensure and establish structures for participation by communities affected by the issue being studied, representatives of organizations, and researchers in all aspects of the research process to improve health and well-being through taking action, including social change” [34]) has been identified as a potential method that can be used to rebuild trust and ensure mutually beneficial research products [32,35,36]. Future studies should use community-based participatory research to build trust, increase participation, and create beneficial outcomes for communities and researchers by co-creating research questions, methods, and recruitment material to increase the likelihood of participation and generalizable results.

Another limitation of this study is the aggregation of NHPI participants, potentially causing measurement invariance in the historical trauma scales. NHPI participants had a mean score of 13.7 (*SD* = 9.0) on the historical loss scale. In a previous study of Native Hawaiian college students, the average historical loss score was 21.4 (*SD* = 9.9) [11]. Although these mean scores do not align (likely due to the sample size and population sampled), they do share similar standard deviations, showing similar variation amongst the sample. Additionally, the historical traumatic events and historical loss scales used in this study were adapted for Native Hawaiian populations. However, NHPI participants were grouped together for this study due to the limited sample size. The umbrella term NHPI represents a number of groups (e.g., Native Hawaiian, Samoan, Tahitian, etc.). These groups, though they share similar experiences of colonialism, possess distinct histories, both in terms of specific events and the timeline of these events. Therefore, the NH adapted historical trauma scales may not accurately capture the experiences of other PI groups. Efforts have been made to develop and adapt historical trauma measures to be used across different Indigenous groups. A recent study examined the psychometric properties of the adapted historical loss scale and Historical Loss Associated Symptoms Scale for Native Hawaiian populations [37]. Both scales were adapted from the original measure developed for use in American Indian and Alaskan Native populations and include items that were screened by Native Hawaiian community members (e.g., distrust, resentment, or fear toward White people; the destruction or damage of traditional food). The results supported the use of both scales with Native Hawaiian populations. Future studies should look to understand the unique historical traumatic experiences of these different Pacific Islander groups to further aid in the development of historical trauma measurement scales. 

The strictly quantitative nature of this study is another limitation. Due to resource restraints, data collection for this project included only quantitative surveys. Although this provided valuable insights into this under-researched field, it provides a limited understanding of the factors that do impact houseless NHPIs well-being and experience of historical trauma. The inclusion of a qualitative section, where participants could provide feedback on the process or talk through the quantitative results, could have provided rich context for these findings. Future studies should consider a mixed-method approach, especially as this field of research develops, to both give voice to participants and provide a more thorough understanding of this area of study.

## 5. Conclusions

This study examined the interaction between NHPI identity and experiences of houselessness on perceived stress and historical trauma. It is important to note that the analyses revealed that the experience of houselessness and NHPI identity did not affect perceived stress levels. Despite the incredibly taxing experience of houselessness, and the historical and modern-day inequities faced by NHPIs, these populations continue to thrive despite these extenuating circumstances, highlighting the true resiliency of these communities. It is important to identify the strengths that NHPI communities possess, which promote resilience in the face of wide-scale crises. The identification of protective factors in NHPI communities, such as family and cultural connection, could help to build community capacity and help clinicians to capitalize on this resilience when working with NHPI populations. It could also be used in the creation of prevention and intervention programs, which decrease the occurrence of adverse health outcomes and promote well-being. This is important to note, as the creation of effective and culturally relevant preventive and intervention programs is essential for decreasing health disparities.

## Figures and Tables

**Table 1:** Participant demographics.

Variable	Response Options	*n* (*%*)
Age	Range	18–24
	Mean	21.37
	Standard Deviation	1.93
Race and Ethnicity ^a^	American Indian or Alaskan Native	2 (3.9%)
	Asian	30 (58.8%)
	Black or African American	4 (7.8%)
	Latinx or Hispanic	10 (19.6%)
	Native Hawaiian or Pacific Islander	26 (51.0%)
	White or European American	25 (49.0%)
	Other	4 (7.8%)
Housing Status	Emergency Shelter	12 (23.5%)
	Transitional Shelter	5 (9.8%)
	Outdoor Public Area	2 (3.9%)
	Living with family; Temporary	8 (15.7)
	Permanent Housing	24 (47.1%)
Gender Identity ^b^	Woman/female	32 (62.7%)
	Man/male	15 (29.4%)
	Gender non-conforming	2 (3.9%)
	Prefer not to answer	1 (2.0%)
Education ^b^	Less than High School	3 (5.9%)
	Some High School	4 (7.8%)
	High School Diploma or GED	14 (27.5%)
	Some College	9 (17.6%)
	Associate degree/Vocational	7 (13.7%)
	4-year College Degree	7 (13.7%)
	Graduate Degree	3 (5.9%)
Income ^b^	$0 to $15,000	21 (41.2%)
	$15,001 to $20,000	5 (9.8%)
	$20,001 to $25,000	4 (7.8%)
	$25,001 to $30,000	1 (2.0%)
	$30,001 to $35,000	2 (3.9%)
	$40,001 to $45,000	1 (2.0%)
	$45,001 to $50,000	1 (2.0%)
	$55,001 to $60,000	1 (2.0%)
	$60,001 to $65,000	1 (2.0%)
Experience of Food Insecurity ^b^	Daily	7 (13.7%)
	More than once a week	10 (19.6%)
	More than once a month	9 (17.6%)
	Never	20 (39.2%)
Access to Mental Health Services ^b^	Yes	32 (62.7%)
	No	16 (31.4%)

Note. *N* = 51. Participants selected from response options provided. ^a^ Total will be greater than 100% as participants were able to select multiple fields. ^b^ Missing data.

## Data Availability

The data presented in this study are available on request from the corresponding author due to their containing information that could compromise the privacy of research participants.
